# Supplementary material for: Label-free imaging for quality control of cardiomyocyte differentiation
Source: Nat Commun. 2021 Jul 28;12:4580. doi: 10.1038/s41467-021-24868-1 (PMC8319125; doi:10.1038/s41467-021-24868-1)
Supplement: Supplementary file 1 — Supplementary information [file 41467_2021_24868_MOESM1_ESM.pdf]

Supplementary information for

label-free imaging for quality control of cardiomyocyte differentiation

Tongcheng Qian[1,#,*], Tiffany M. Heaster[1,2,#], Angela R. Houghtaling[1], Kexin Sun[1], Kayvan Samimi[1], Melissa C. Skala[1,2,*]

[1]Morgridge Institute for Research, Madison, WI, 53715, USA; [2]Department of Biomedical Engineering, University of Wisconsin-Madison, Madison, WI, 53706, USA

[#]These authors contribute equally to this work.

[*]Correspondence should be addressed to T.Q (tqian5@wisc.edu) or M.C.S (mcskala@wisc.edu).

Supplementary figures and figure legends

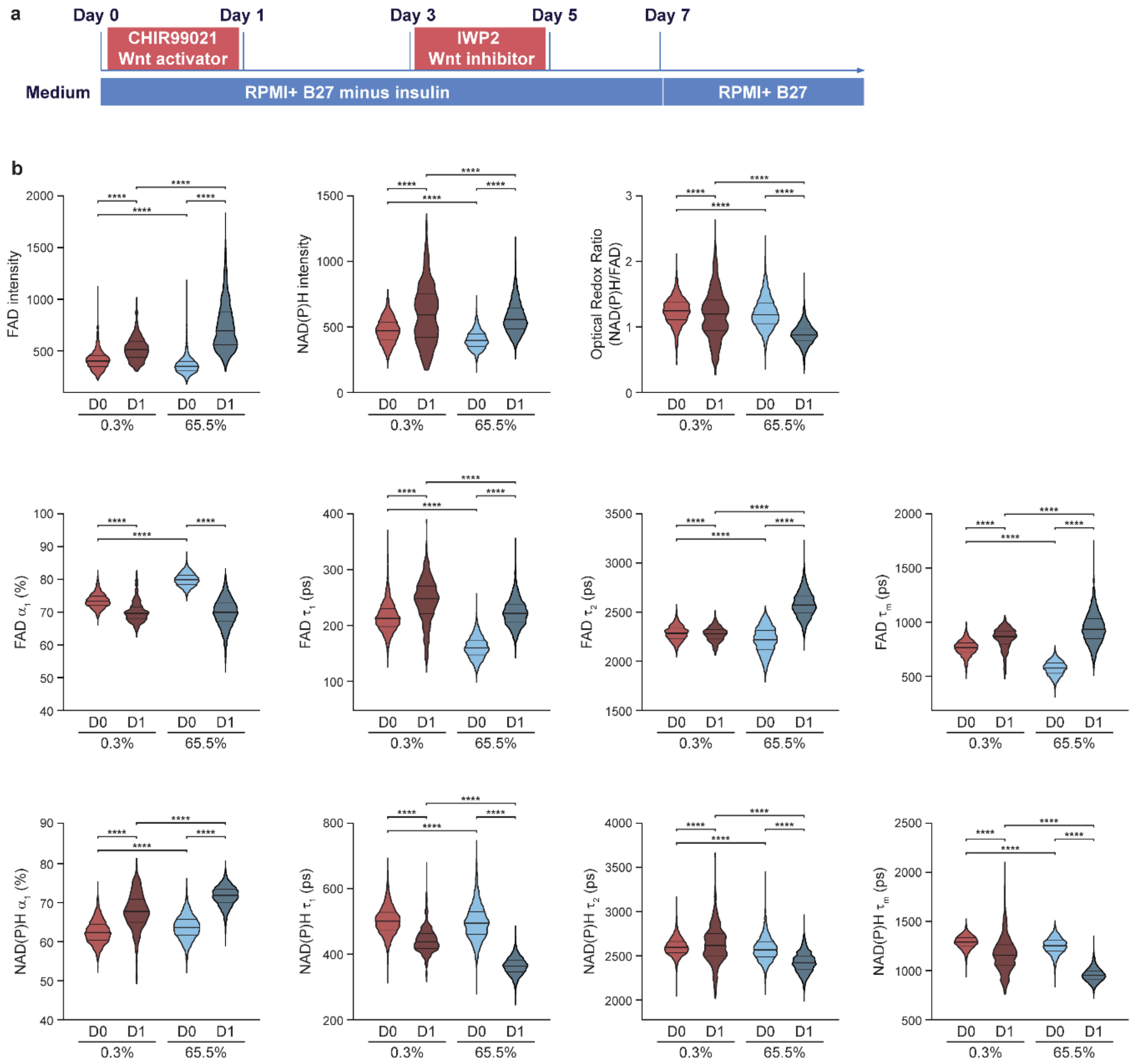

Supplementary Figure 1. NAD(P)H and FAD fluorescence change differently in the first 24 hours for cells in low vs. high cardiomyocyte differentiation efficiency conditions. (a) Cardiomyocyte differentiation protocol from day 0 to day 7. **(b)** hPSCs were differentiated into CMs following an established method[11]. Single-cell quantitative analysis of OMI variables, including FAD intensity, NAD(P)H intensity, optical redox ratio, FAD α_1, τ_1, τ_2, τ_m, and NAD(P)H α_1, τ_1, τ_2, τ_m, for low differentiation (0.3% cTnT+ on day 12) and high differentiation (65.5% cTnT+ on day 12) efficiencies on day 0 ("D0", immediately pre-treatment) and day 1

("D1", 24 hours post-treatment with CHIR99021). n = 2458, 633, 3,534, and 4446 cells for 0.3% D0, 0.3% D1, 65.5% D0, and 65.5% D1, respectively. On differentiation day 12, cells were verified by flow cytometry with cTnT labelling. Data are presented in violin plots with middle bar for the mean, lower bar for 25th percentile, and upper bar for 75th percentile. Statistical significance was determined by one-way analysis of variance (ANOVA) followed by Tukey's post hoc tests. ****$p < 0.0001$. ps, picoseconds.

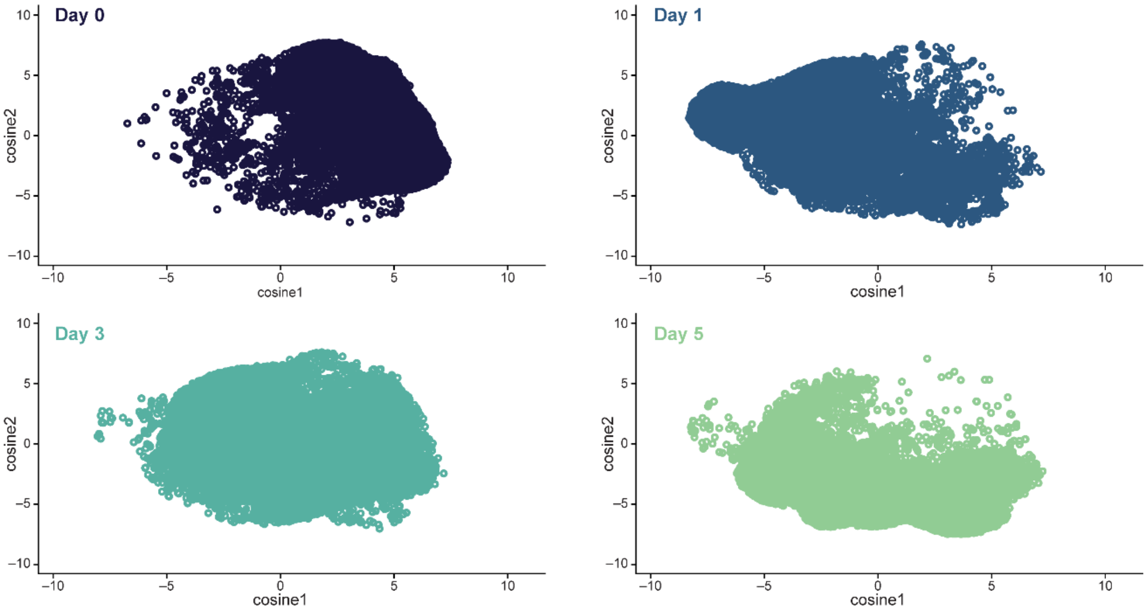

Supplementary Figure 2. UMAP plotted by separate days and conditions. Uniform Manifold Approximation and Projection (UMAP) dimensionality reduction was performed on all 13 autofluorescence variables (optical redox ratio, NAD(P)H τ_m, τ_1, τ_2, α_1, α_2, and intensity; FAD τ_m, τ_1, τ_2, α_1, α_2, and intensity) for each cell and projected onto 2D space. Cells from all 11 conditions shown in Table 1 are plotted by days separately. Each dot represents one single cell, and n = 25304, 25470, 26228, and 23484 cells for day 0, 1, 3, and 5, respectively.

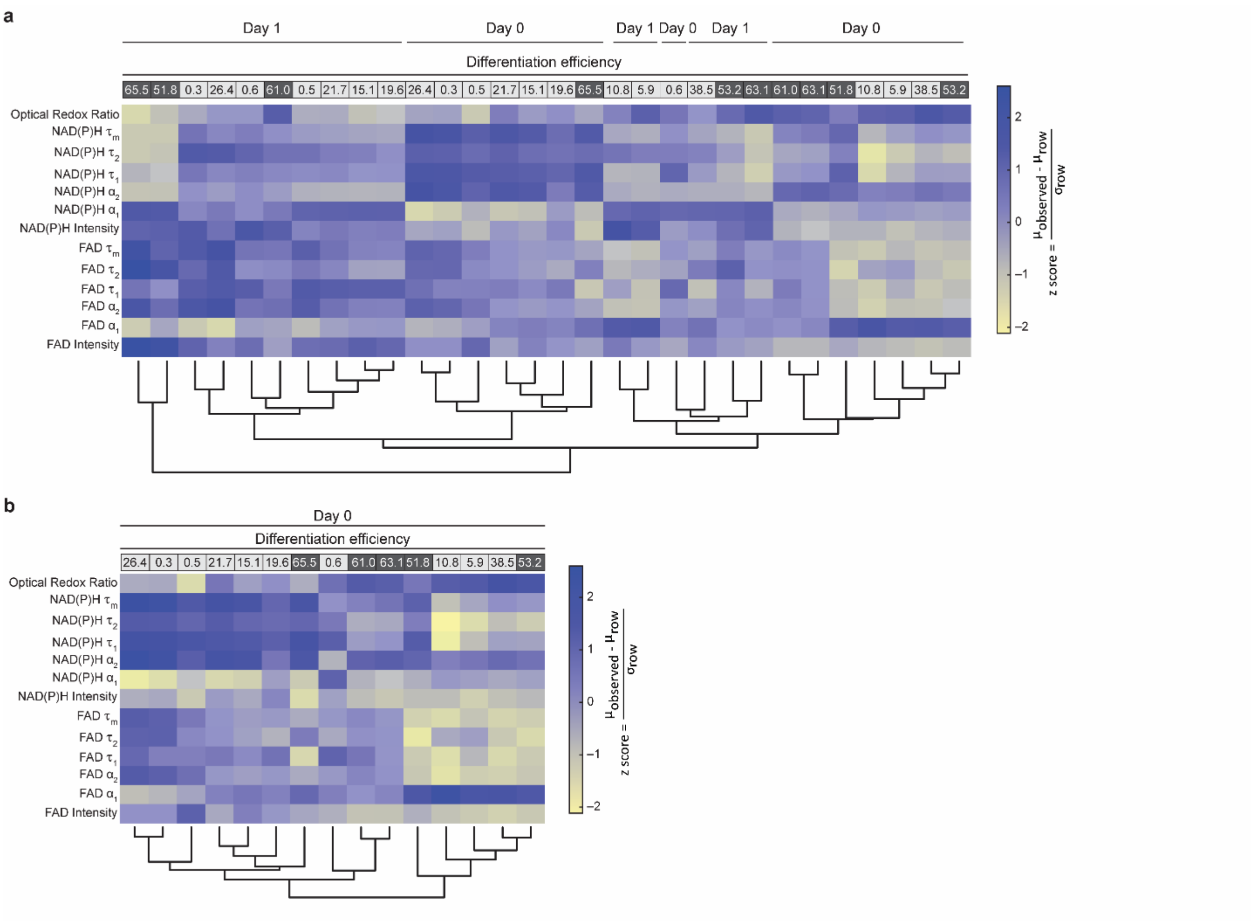

Supplementary Figure 3. Heatmap dendrogram clustering of OMI variable z-score on day 0 and day 1. Heatmap dendrogram clustering based on similarity of average Euclidean distances across all OMI variable z-scores was performed on (**a**) day 0 (immediately pre-treatment) and day 1 cells (24 hours post-treatment with CHIR99021) across all 15 conditions together or (**b**) day 0 alone. Conditions are indicated by the CM differentiation efficiency percentages as noted by column labels at the top of the heatmap (quantified by flow cytometry cTnT+ on day 12, full conditions given in Table 1). Low differentiation efficiencies (< 50%) are shaded in light gray and high differentiation efficiencies are shaded in dark gray (≥ 50%). Z-score = $\frac{\mu_{observed}-\mu_{row}}{\sigma_{row}}$, where $\mu_{observed}$ is the mean value of each variable for each condition, μ_{row} is the mean value of each variable for all 15 conditions together, and σ_{row} is the standard deviation of each variable

across all 15 conditions. Autofluorescence variables are indicated on the left side as row labels. n = 28590 and 30463 cells for day 0, 1, respectively.

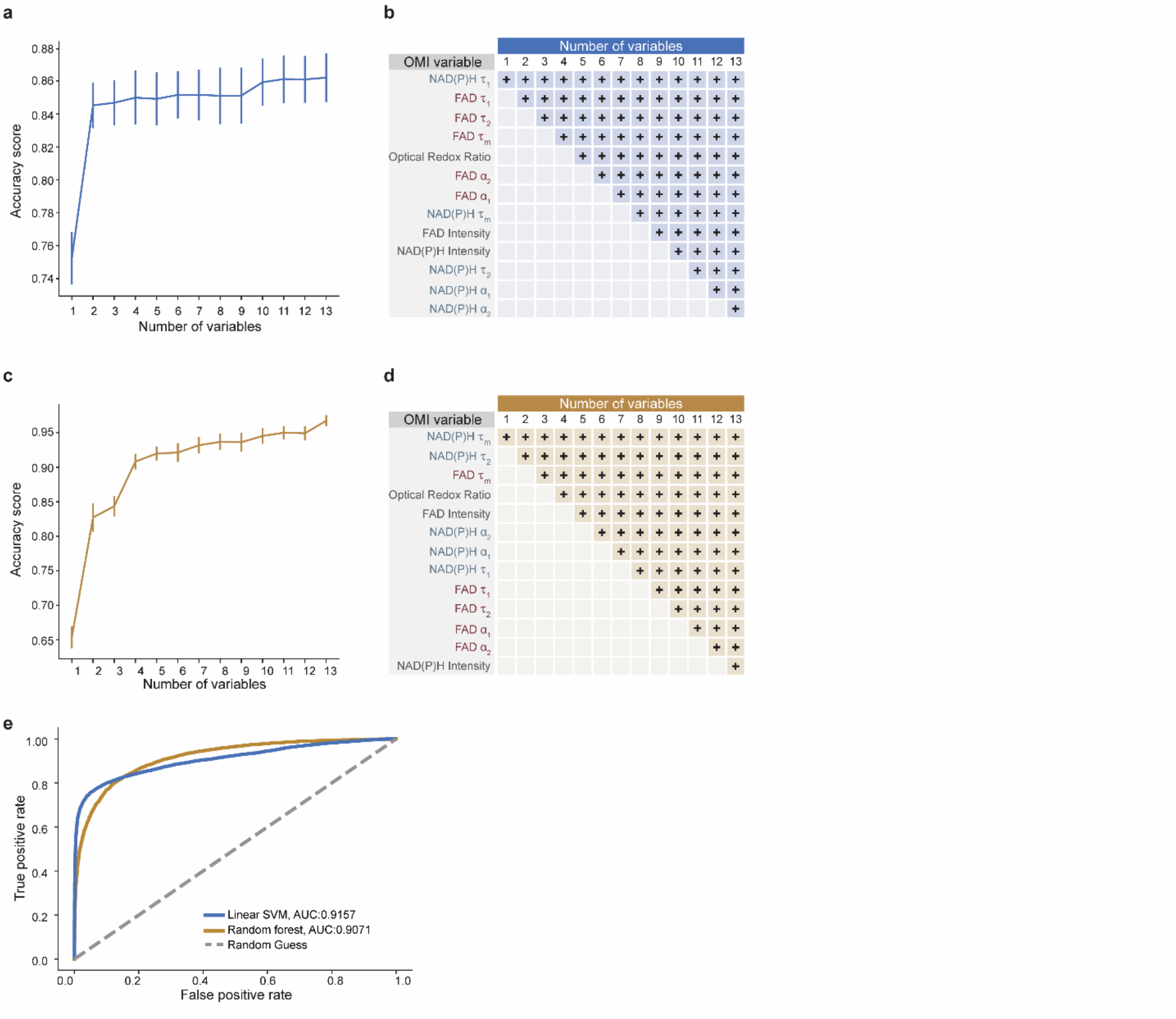

Supplementary Figure 4. Support vector machine and random forest models for classification of cells on day 1. All OMI data (optical redox ratio, NAD(P)H τ_m, τ_1, τ_2, α_1, α_2, and intensity; FAD τ_m, τ_1, τ_2, α_1, α_2, and intensity) from day 1 cells separated into two datasets. Dataset 1was randomly partitioned into training and validation datasets for proportions of 80%

and 20%, respectively (n = 8974 cells for training, 2244 cells for test). Dataset 2 was used for evaluation of classifier performance. Classification accuracy with respect to number of OMI variables was evaluated by chi-squared variable selection to separate low (< 50% cTnT+ on day 12) and high (≥ 50% cTnT+ on day 12) differentiation efficiency conditions by (**a**) support vector machine (SVM). (**b**) The variables included for each SVM [specified by numbers of variables on the x-axis in (**a**)]. The OMI variables included in each instance (e.g., 3, 4) are indicated by a blue + in each column. (**c**) Classification accuracy with respect to number of OMI variables for random forest classification. (**d**) The variables included for each random forest classifier [specified by numbers of variables on the x-axis in (**c**)]. The accuracy scores are presented as mean ± STDEV. The OMI variables included in each instance are indicated by a yellow + in each column (**e**) Model performance was evaluated by receiver operating characteristic (ROC) curves displaying classification performance with the SVM (blue curve) or random forest model (yellow curve) using all 13 OMI variables. The area under the curve (AUC) is provided for SVM and random forest classifiers as indicated in the legend.

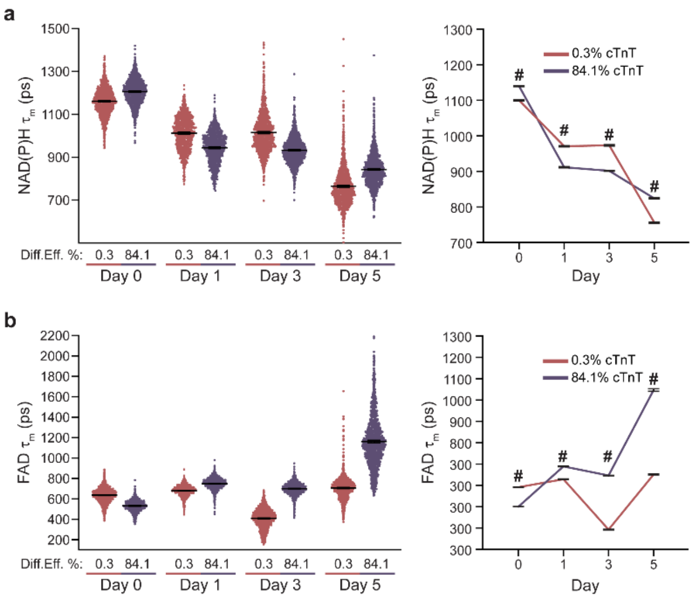

Supplementary Figure 5. **Changes in lifetimes of NAD(P)H and FAD during the first 5-days of differentiation**. Single-cell quantitative analysis of mean lifetimes (τ_m) of (**a**) NAD(P)H and (**b**) FAD on days 0, 1, 3, and 5. Unpaired two-tailed student T-test was performed between low (0.3% cTnT+) and high (84.1% cTnT+) differentiation efficiency conditions on each day. #p<0.0001 for 0.3% vs. 84.1% differentiation efficiency conditions on day 0, day 1, day 3, and day 5, respectively. Data were collected from over 500 cells for each day for each condition and presented as dot plots with bars for the mean and 95% CI, and the line graph as mean ± SEM. ps, picoseconds.

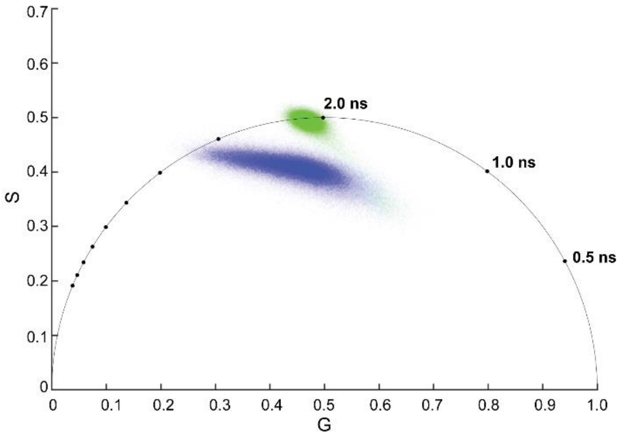

Supplementary Figure 6. Representative phasor plots reveal separation of NAD(P)H and EGFP decays. Phasor plots of fluorescence decays provide a visual distribution of the molecular species in an image by clustering pixels with similar lifetimes, which allows assessment of overlap between lifetime species. The phasor fluorescence decay plots are derived from a Fourier transformation of the fluorescence lifetime decay[46]. The fluorescence lifetime of each pixel in the image is presented in the 2D phasor plot with the unitless horizontal (G) and vertical (S) axes. Blue dots are NAD(P)H decays and green dots are EGFP decays. The lifetimes of 3 positions on the unit circle are given in nanoseconds for reference.

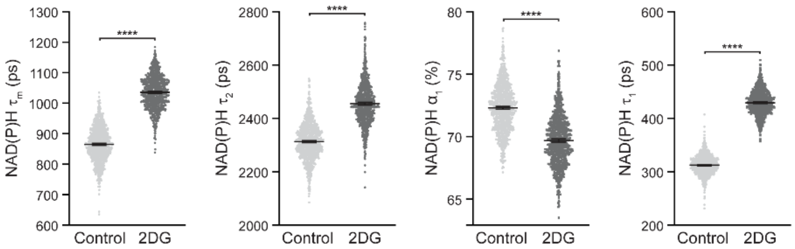

Supplementary Figure 7. Changes of NAD(P)H lifetime variables after treatment with 2DG. Single-cell quantitative analysis of NAD(P)H lifetime variables (τ_m, τ_2, α_1, τ_1) for H9 embryonic stem cells before and 2 hours after 10 mM 2DG treatment. n = 1051 and 900 cells for before and after 2DG treatment, respectively. Data are presented as dot plots with bars for the mean and 95% CI. Statistical significance was determined by unpaired two-tailed Student's T-test. ****$p<0.0001$. ps, picoseconds.
