# Supplementary material for: Label-free imaging for quality control of cardiomyocyte differentiation
Source: Nat Commun. 2021 Jul 28;12:4580. doi: 10.1038/s41467-021-24868-1 (PMC8319125; doi:10.1038/s41467-021-24868-1)
Supplement: Supplementary file 2 — Reporting Summary [file 41467_2021_24868_MOESM2_ESM.pdf]

nature research

Corresponding author(s): Tongcheng Qian and Melissa Skala

Last updated by author(s): Jun 29, 2021

Reporting Summary

Nature Research wishes to improve the reproducibility of the work that we publish. This form provides structure for consistency and transparency in reporting. For further information on Nature Research policies, see our Editorial Policies and the Editorial Policy Checklist.

Statistics

For all statistical analyses, confirm that the following items are present in the figure legend, table legend, main text, or Methods section.

n/a	Confirmed	
☐	☒	The exact sample size (n) for each experimental group/condition, given as a discrete number and unit of measurement
☐	☒	A statement on whether measurements were taken from distinct samples or whether the same sample was measured repeatedly
☐	☒	The statistical test(s) used AND whether they are one- or two-sided<br>*Only common tests should be described solely by name; describe more complex techniques in the Methods section.*
☐	☒	A description of all covariates tested
☐	☒	A description of any assumptions or corrections, such as tests of normality and adjustment for multiple comparisons
☐	☒	A full description of the statistical parameters including central tendency (e.g. means) or other basic estimates (e.g. regression coefficient) AND variation (e.g. standard deviation) or associated estimates of uncertainty (e.g. confidence intervals)
☐	☒	For null hypothesis testing, the test statistic (e.g. F, t, r) with confidence intervals, effect sizes, degrees of freedom and P value noted<br>*Give P values as exact values whenever suitable.*
☒	☐	For Bayesian analysis, information on the choice of priors and Markov chain Monte Carlo settings
☐	☒	For hierarchical and complex designs, identification of the appropriate level for tests and full reporting of outcomes
☒	☐	Estimates of effect sizes (e.g. Cohen's d, Pearson's r), indicating how they were calculated

Our web collection on statistics for biologists contains articles on many of the points above.

Software and code

Policy information about availability of computer code

Data collection	Data was collected using Prairie View 5.4, FACSCalibur flow cytometer (BD Biosciences)
Data analysis	Data was analyzed using commercial and open-source softwares: SPCImage(from 6.0-8.0), Python (v3.7.3), R (v3.6.2), ImageJ (from 1.50g-1.53j), CellProfiler(3.0.0), and FlowJo. (from 7.0-10.7)

For manuscripts utilizing custom algorithms or software that are central to the research but not yet described in published literature, software must be made available to editors and reviewers. We strongly encourage code deposition in a community repository (e.g. GitHub). See the Nature Research guidelines for submitting code & software for further information.

Data

Policy information about availability of data

All manuscripts must include a data availability statement. This statement should provide the following information, where applicable:

- Accession codes, unique identifiers, or web links for publicly available datasets
- A list of figures that have associated raw data
- A description of any restrictions on data availability

The main data supporting the results in this study are available within the paper and its Supplementary Information. The raw and analyzed datasets generated during the study are too large to be publicly shared, however, they are available for research purposes from the corresponding authors on reasonable request. We also included analyzed autofluorescence data together with computational codes. All files used to generate and support the results from the associated code have been deposited in the following repository: https://github.com/skalalab/cardiomyocyte_differentiation.

April 2020

Field-specific reporting

Please select the one below that is the best fit for your research. If you are not sure, read the appropriate sections before making your selection.

- [x] Life sciences
- [ ] Behavioural & social sciences
- [ ] Ecological, evolutionary & environmental sciences

For a reference copy of the document with all sections, see nature.com/documents/nr-reporting-summary-flat.pdf

Life sciences study design

All studies must disclose on these points even when the disclosure is negative.

Sample size	For autofluorescence imaging, 2-3 independent cultures were imaged with 4-6 images for each condition yielding 500-5000 cells. The number of cells for each condition has been provided in the figure legend. For flow cytometry, 20000 events were collected for each sample. 3 independent samples were collected for each condition. The replicates and sample size were based on our previous studies by using single cell OMI data and preliminary data for statistical power.
Data exclusions	No data was excluded
Replication	Data were acquired from multiple independent replicates and has been indicated in the manuscript. All attempts at replication were successful. Independent replicates were performed more than two weeks apart. Independent cell lines were evaluated over two years apart.
Randomization	All OMI data from day 1 cells were randomly partitioned into training and test datasets using 15-fold cross validation, with training and test proportions of 80% and 20%, respectively
Blinding	Imaging and differentiation were performed by T.Q. Single cell analysis was performed by A.R.H and K.S. Multivariate analysis and classification were performed by T.M.H.

Reporting for specific materials, systems and methods

We require information from authors about some types of materials, experimental systems and methods used in many studies. Here, indicate whether each material, system or method listed is relevant to your study. If you are not sure if a list item applies to your research, read the appropriate section before selecting a response.

Materials & experimental systems

n/a	Involved in the study	
[ ]	[x]	Antibodies
[ ]	[x]	Eukaryotic cell lines
[x]	[ ]	Palaeontology and archaeology
[x]	[ ]	Animals and other organisms
[x]	[ ]	Human research participants
[x]	[ ]	Clinical data
[x]	[ ]	Dual use research of concern

Methods

n/a	Involved in the study	
[x]	[ ]	ChIP-seq
[ ]	[x]	Flow cytometry
[x]	[ ]	MRI-based neuroimaging

Antibodies

Antibodies used	anti-cTnT (ThermoScientific, catlog: MS-295-P1, Lab Vision; 1:200) and secondary antibody (Thermo Fisher; goat anti-mouse, Alexa Fluor 488; 1:500)
Validation	Antibody validated as noted on the manufacturer's website. Anti-cTnT was validated using commercially available iPSC-derived cardiomyocytes as positive control and hiPSC as negative control. The dilution of the primary antibody was adopted from the instruction http://tools.thermofisher.com/content/sfs/brochures/D11736~.pdf provided by thermoscientific.

Eukaryotic cell lines

Policy information about cell lines

Cell line source(s)	WA09, WA13, IMR90-4, and 19-9-11 were purchased from Wicell. NKX2.5EGFP/+ hPSC (ES03) was gifted from a previous collaborator (Sean Palecek's lab at the UW-Madison). No federal funding was involved in this project with embryonic stem cell lines.
Authentication	WA09, WA13, IMR90-4, and 19-9-11 were purchased from Wicell and authenticated by Wicell with MTAs.

Mycoplasma contamination	Cell lines were regularly tested for mycoplasma through Wicell service. All cell lines used in the experiments are mycoplasma negative.
Commonly misidentified lines (See ICLAC register)	No commonly misidentified cell lines were used.

Flow Cytometry

Plots

Confirm that:

- [x] The axis labels state the marker and fluorochrome used (e.g. CD4-FITC).
- [x] The axis scales are clearly visible. Include numbers along axes only for bottom left plot of group (a 'group' is an analysis of identical markers).
- [x] All plots are contour plots with outliers or pseudocolor plots.
- [x] A numerical value for number of cells or percentage (with statistics) is provided.

Methodology

Sample preparation	Cells on differentiation day 12 were dissociated with Accutase, fixed in 1% PFA for 15 minutes at room temperature, and then blocked with 0.5% bovine serum albumin (BSA) with 0.1% Triton X-100. Cells were then stained with primary antibody anti-cTnT (Lab Vision; 1:200) and secondary antibody (Thermo Fisher; goat anti-mouse, Alexa Fluor 488; 1:500) in 0.5% BSA with 0.1% Triton X-100.
Instrument	Data were collected on a FACSCalibur flow cytometer
Software	Data were analyzed with FlowJo
Cell population abundance	Each sample was run to collect over 20000 events based on previous published references. Previous published references showed that over 5000 events is enough for cell population assay. Post-sorting was not applied.
Gating strategy	Populations were gated based on the negative control and clear separation of cTnT expression level from high and low differentiation efficiencies. Gating strategy is provided in main figure.

- [x] Tick this box to confirm that a figure exemplifying the gating strategy is provided in the Supplementary Information.
